# Crew’s Mustard Plaster

**Published:** 1868-05

**Authors:** 


Crew's Mustard Plaster.—Improvements in the minor matters pertaining to the sick room are often practically the most important. There are few physicians who, on the simple application of a sinapism, have not met with vexatious delays and disappointment, owing to the inertness of the article used. The mustard of the shops is almost universally an adulteration, as well as the larger proportion of that sold at drug stores. A fair trial of Crew's Spread Mustard Plaster proves it to be perfectly reliable, and superior to either M. Riggollet's "Papier Sinapisé" or "Cooper's Sinapiæ Tissue," which owe their virtue to Cayenne pepper. Crew's plasters are made from the best English mustard and warranted to retain their qualities for an indefinite period.

